# purgeR: inbreeding and purging in pedigreed populations

**DOI:** 10.1093/bioinformatics/btab599

**Published:** 2021-08-18

**Authors:** Eugenio López-Cortegano

**Affiliations:** Institute of Evolutionary Biology, School of Biological Sciences, University of Edinburgh, Edinburgh EH9 3FL, UK

## Abstract

**Summary:**

Inbreeding depression and genetic purging are important processes shaping the survivability and evolution of small populations. However, detecting purging is challenging in practice, in part because there are limited tools dedicated to it. I present a new R package to assist population analyses on detection and quantification of the inbreeding depression and genetic purging of biological fitness in pedigreed populations. It includes a collection of methods to estimate different measurements of inbreeding (Wright’s, partial and ancestral inbreeding coefficients) as well as purging parameters (purged inbreeding, and opportunity of purging coefficients). Additional functions are also included to estimate population parameters, allowing to contextualize inbreeding and purging these results in terms of the population demographic history. purgeR is a valuable tool to gain insight into processes related to inbreeding and purging, and to better understand fitness and inbreeding load evolution in small populations.

**Availability and implementation:**

purgeR is an R package available at CRAN, and can be installed via install.packages(“purgeR”). Source code is maintained at a GitLab repository (https://gitlab.com/elcortegano/purgeR).

**Supplementary information:**

Supplementary data are available at *Bioinformatics* online.

## 1 Introduction

Inbreeding may result in the decline of biological fitness due to the increase in the frequency of homozygote genotypes for deleterious recessive alleles, causing the so-called ‘inbreeding depression’ (Charlesworth and Charlesworth, 1987). However, as deleterious alleles become more exposed under inbreeding, selection also becomes more effective removing, or ‘purging’, them (Charlesworth, 2018; Glémin, 2003). Consequently, the expectation of population fitness evolution under inbreeding changes with purging, possibly allowing for the survival of small populations (Hedrick and García-Dorado, 2016).

Pedigree information has attracted the attention of genetic purging models, as these allow for direct inferences on single individuals based on their genealogical history, and many conservation programs maintain studbooks with pedigree records. Boakes and Wang (2005) used ancestral inbreeding coefficients (*F_a_*, Ballou, 1997) to measure the consequences of purging on fitness, given the expectation that individuals born from more inbred ancestors are expected to be more fit than individuals with the same level of inbreeding but less inbred ancestors. Gulisija and Crow (2007) developed a method to evaluate the potential reduction in the individual inbreeding load (*B*) using the probability of transmission of highly deleterious recessive alleles under inbreeding. García-Dorado (2012) defined a purged inbreeding coefficient (*g*) that measures the expected frequency of recessive deleterious loci in homozygosity, as a function of a purging coefficient (*d*) that relates to the recessive component of deleterious effects.

While many software packages have been developed to compute inbreeding, resources for purging analysis are more limited. To my knowledge, *F_a_* is only computed by a few software packages (Baumung *et al.*, 2015; Doekes *et al.*, 2020; García-Dorado *et al.*, 2016), and only PURGd estimates *g* (García-Dorado *et al.*, 2016). No informatic tool is available to compute Gulisija and Crow’s parameters for the opportunity of purging. purgeR computes all these parameters and others, including parameters related to population diversity and demography (e.g. effective population size, *N_e_*), all functions being introduced in tutorials accessible via browseVignettes (“purgeR”).

## 2 Input data

For illustrative purposes here, a population with known fitness and *B *=* *4.4 was simulated with size *N *=* *10^3^ for 10^3^ generations and then bottlenecked to *N *=* *25 for 50 generations using SLiM 3.5 (Haller and Messer, 2019), under conditions favorable to the detection of purging, similarly as in García-Dorado *et al.* (2016). Details on the mutational model used and code to reproduce the simulation are given in Supplementary File S1. The simulated pedigree is included as Supplementary Table S1. Input pedigrees are required to be ‘data.frame’ objects in R, and to include individual, maternal and paternal identities.

## 3 Opportunity of purging

Here, the computation of opportunity of purging measures is presented, since this is the major novelty in purgeR compared to its predecessor PURGd (from which many functions are reimplemented), apart from other improvements related to portability, performance and usability. Some assays on the performance of purgeR are given in Supplementary File S2.

Total (*O*) and expressed (*O_e_*) opportunity of purging can be computed for every individual to provide an estimate of the expected reduction in *B*. While *O* measures the potential reduction of *B* as a consequence of having inbred ancestors, *O_e_* relates to the reduction in expressed *B* as a consequence of having inbred ancestors, and being homozygous for alleles derived from them (Gulisija and Crow, 2007). They can be computed in simple pedigrees for an individual *i* as:

$$O_{\left(i\right)}=\sum_{j}^{}\sum_{k}^{}{\left(1/2\right)}^{n-1}F_{j}$$

 

$$O_{e(i)}=\sum_{j}^{}2F_{i(j)}F_{j}$$

where the summation *j* is over all inbred ancestors, and the summation *k* is over all paths from *i* to ancestor *j* (each involving a number of individuals *n*, *i* and *j* included). *F_i(j)_* is the partial inbreeding of *i* referred to ancestor *j*, indicating *i’*s probability to be inbred by descent for alleles derived from *j*. *O* and *O_e_* are computed via the function purgeR::ip_op(), and notes on the validation for this function and others in the package are provided in Supplementary File S3.

For complex pedigrees involving several autozygotes in the same path, these parameters need to be corrected by discounting from close ancestors’ contributions the contribution made by far ancestors (Gulisija and Crow, 2007). However, equations for complex pedigrees derived by Gulisija and Crow involve highly nested complex loops and recursivity, and are not scalable. To make this method more usable, a heuristic approach was developed to correct *O* and *O_e_* measurements. This approach skips far ancestors, that contribute little to *O* and *O_e_*, and also to its correction terms. Specifically, given an individual *i* of interest, contributions to *O_i_* and *O_e(i)_* from far ancestors *k* are ignored as long as *F_j(k)_* > 0, where *j* is an intermediate ancestor. As a drawback, this method can result in estimates of *O *>* *1 and *O_e_* > *F*, particularly in cases involving many recent ancestors such that *F_j(k)_* = 0 (e.g. from consecutive generations) undergoing selfing or breeding very close relatives. These situations are unexpected under the original model, and in these cases *O *=* *1 and *O_e_ *=* F* should be considered.

**Fig. 1.**
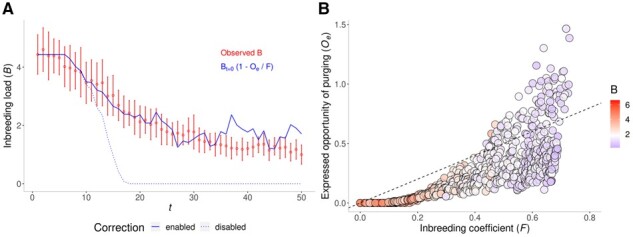
Inbreeding load and opportunity of purging. (**A**) Observed decline of *B* over generations (in read, mean values as points and error bars spanning one standard deviation). Expected *B* is given in blue lines, calculated as *B_t=0_*(1 - *O_e_*/*F*), where *O_e_*/*F* represents the normalized opportunity of purging (Gulisija and Crow, 2007). The solid line corresponds to corrected *O_e_* estimates, and dotted line to raw *O_e_* values. (**B**) Relationship between *O_e_* and *F*, colored by value of *B* (white is the median pedigree value *B *=* *1.8; increasingly red and blue coloration indicate higher and lower *B* values, respectively). A dashed line indicates the value *O_e_ *=* F*. Code can be found in Supplementary File S4

Figure 1A shows the observed decline of *B* in the bottlenecked population, together with expectations based on normalized *O_e_* estimated from the pedigree (i.e. *O_e_*/*F*), and also based on purged inbreeding. Figure 1B shows the substantial variation in *O_e_* for individuals with the same level of inbreeding. It also shows the problem of obtaining *O_e_*>*F* estimates. However, as illustrated in Figure 1A, assuming *O_e_*=*F* in such cases allows to estimate *B* reasonably well. Results for alternative mutational models, and an example on a real population, are given in Supplementary File S3, and show that using both corrected and uncorrected *O_e_* estimates might be useful setting, respectively, lower and upper bounds to *B* decline.

It must be noted that Guliisja and Crow’s (2007) model assumes highly deleterious and recessive mutations, thus relating to the most deleterious component of the inbreeding load, and ignoring the possible purging of variants with low effect sizes. Evidence from genomic studies however suggest that purging is only relevant for highly deleterious variants (Grossen *et al.*, 2020). In small populations affected by drift, purging is also expected to be efficient only for strongly deleterious alleles (Glémin, 2003). Therefore, assumptions of the model should hold in most practical cases. In addition, estimation of inbreeding load decline using *O* and *O_e_* provide a way to estimate inbreeding load decline that is not dependent on accurate measures of fitness and other factors, which can be troublesome or be incomplete in many real scenarios.

## 4 Concluding remarks

purgeR is a versatile toolkit to measure inbreeding and purging parameters in pedigreed populations. The inclusion of opportunity of purging parameters is a valuable contribution to the field, since it allows for the inference of purging without requiring more information than the pedigree structure.

## Supplementary Material

btab599_Supplementary_Data

## References

[btab599-B1] Ballou J.D. (1997) Ancestral inbreeding only minimally affects inbreeding depression in mammalian populations. J. Hered., 88, 169–178. 9183845 10.1093/oxfordjournals.jhered.a023085

[btab599-B2] Baumung R. et al (2015) GRAIN: a computer program to calculate ancestral and partial inbreeding coefficients using a gene dropping approach. J. Anim. Breed. Genet., 132, 100–108. 25823836 10.1111/jbg.12145

[btab599-B3] Boakes E.H. , Wang J. (2005) A simulation study on detecting purging of inbreeding depression in captive populations. Genet. Res., 86, 139–148. 16356287 10.1017/S001667230500772X

[btab599-B4] Charlesworth B. (2018) Mutational load, inbreeding depression and heterosis in subdivided populations. Mol. Ecol., 27, 4991–5003. 30415505 10.1111/mec.14933

[btab599-B5] Charlesworth D. , Charlesworth B. (1987) Inbreeding depression and its evolutionary consequences. Annu. Rev. Ecol. Syst., 18, 237–268.

[btab599-B6] Doekes H.P. et al (2020) Revised calculation of Kalinowski’s ancestral and new inbreeding coefficients. Diversity, 12, 155.

[btab599-B7] García-Dorado A. (2012) Understanding and predicting the fitness decline of shrunk populations: inbreeding, purging, mutation, and standard selection. Genetics, 190, 1461–1476. 22298709 10.1534/genetics.111.135541 PMC3316656

[btab599-B8] García-Dorado A. et al (2016) Predictive model and software for inbreeding-purging analysis of pedigreed populations. G3, 6, 3593–3601. 27605515 10.1534/g3.116.032425 PMC5100858

[btab599-B9] Glémin S. (2003) How are deleterious mutations purged? Drift versus nonrandom mating. Evolution, 57, 2678–2687. 14761049 10.1111/j.0014-3820.2003.tb01512.x

[btab599-B10] Grossen C. et al (2020) Purging of highly deleterious mutations through severe bottlenecks in Alpine ibex. Nat. Commun., 11, 1001. 32081890 10.1038/s41467-020-14803-1 PMC7035315

[btab599-B11] Gulisija D. , Crow J.F. (2007) Inferring purging from pedigree data. Evolution, 61, 1043–1051. 17492959 10.1111/j.1558-5646.2007.00088.x

[btab599-B12] Haller B.C. , Messer P.W. (2019) SLiM 3: forward genetic simulations beyond the Wright-Fisher model. Mol. Biol. Evol., 36, 632–637. 30517680 10.1093/molbev/msy228 PMC6389312

[btab599-B13] Hedrick P.W. , García-Dorado A. (2016) Understanding inbreeding depression, purging, and genetic rescue. Trends Ecol. Evol., 31, 1109–1144. 10.1016/j.tree.2016.09.005 27743611

