# Supplementary material for: purgeR: inbreeding and purging in pedigreed populations
Source: Bioinformatics. 2021 Aug 18;38(2):564–5. doi: 10.1093/bioinformatics/btab599 (PMC8723146; doi:10.1093/bioinformatics/btab599)
Supplement: btab599_Supplementary_Data [file btab599_supplementary_data.zip › File S1.pdf]

purgeR: Inbreeding and purging in pedigreed populations

File S1: SLiM configuration files

Base population

A base population of size $N = 10^3$ was maintained over $t = 10^3$ generations following a Fisherian model (implying random mating and discrete generations). The mutational model includes mutations with fixed effects arising at a mutation rate of $\mu = 5 \times 10^{-6}$. Mutations are fully recessive ($h = 0$) and strongly deleterious ($s = 0.3$), favoring the occurrence and detection of purging. The inbreeding load (B) was calculated as $B = 2d \sum_{i=1}^{n} q(1-q)$ over all n loci, where q is the allele frequency and $d = \frac{1}{2}s(1-2h) = 0.15$. The genome is constituted of 1000 freely recombining regions each of 21 bp in length. At the end of the simulation, the base population had $B = 4.4$.

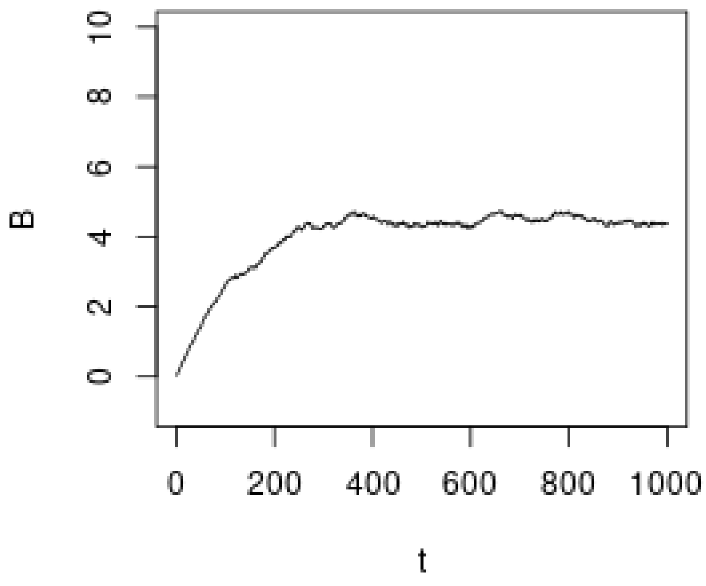

Figure 1: Change in mean inbreeding load (B) on the base population

Note that the base population will be saved in a file named `basepop.trees`. This file will be loaded by the script responsible for simulating the bottlenecked population. To reduce the size of this file, neutral mutations can be disabled.

The following SLiM configuration file was run in SLiM 3.5:

```
initialize() {
    setSeed(1234);
```

```
    defineConstant("h", 0.0);                // degree of dominance
    defineConstant("s", -0.3);               // selection coefficient
    defineConstant("d", -0.5*s*(1.0-2.0*h)); // dominant deviation
    defineConstant("maxB", 10.0);            // maximum inbreeding load
    defineConstant("N", 1000);               // population size
    defineConstant("RSCRIPT", "/usr/bin/Rscript");
    initializeTreeSeq();
    initializeMutationRate(5e-6);
    //initializeMutationType("m1", 0.5, "f", 0.0);
    initializeMutationType("m2", h, "f", s);
    //initializeGenomicElementType("g1", m1, 1.0);
    initializeGenomicElementType("g2", m2, 1.0);
    ends = c();
    rates = c();
    for (i in 0:1000) {
        initializeGenomicElement(g2, i*21, i*21); // originally set as g1 for testing
        initializeGenomicElement(g2, i*21+1, i*21+20);
        ends = c(ends,  i*21, i*21+20);
        rates = c(rates, 0.5, 0.0);
    }
    initializeRecombinationRate(rates, ends);
}

1 {
    sim.addSubpop("p1", N);
    sim.setValue("history", NULL);
    defineConstant("pngPath", writeTempFile("plot_", ".png", "")); // in QtSLiM
    if(exists("slimgui"))
        slimgui.openDocument(pngPath);
}

1: late() {

    q = sim.mutationFrequencies(p1);
    B = 2*q*(1-q)*d; // per locus inbreeding load
    B = sum(B);      // total inbreeding load
    if (B > maxB) {
        print("Inbreeding load is excessively high!");
        sim.simulationFinished();
    }
    catn(B);
    log = c(sim.getValue("history"), B);
    sim.setValue("history", log);
    if (sim.generation % 100 == 0) {
        // R code to plot fitness
        // Every 100 generations, an R script is generated and written
        // then called by Rscript
        rstr = paste(c('{',
            't <- (1:' + size(log) + ')',
            'B <- c(' + paste(log, sep=", ") + ')',
            'png(width=4, height=4, units="in", res=72, file="' + pngPath + '")',
            'plot(x=t, y=B, xlim=c(0, 1000), ylim=c(-1, 10), type="l")',
            'dev.off()',
```

```
            '}'), sep="\n");
        scriptPath = writeTempFile("plot_", ".R", rstr);
        system(RSCRIPT, args=scriptPath);
    }
}
1000 late() {
    sim.treeSeqOutput("basepop.trees");
}
```

Bottlenecked population

The base population (read from `basepop.trees`) is bottlenecked into a population of size $N = 25$ by sampling, and then this population is maintained over $t = 50$ generations. The mutational model and genome structure are set as before. Pedigree information is recorded for all individuals.

Relative fitness is estimated for all individuals and generations, accounting for the number of deleterious alleles that have become fixed in the genome. A base value of intra-generational fitness is first computed as:

$$w_t = (1 - s)^{n_t}$$

Where n_t is the number of deleterious mutations that have fixed in the genome in generation t (these are named 'substitutions' in SLiM). Then, since all mutations are fully recessive, individual phenotypes for fitness (w_i) are simply calculated as:

$$w_i = w_t \times (1 - s)^{n_i}$$

Where n_i is the number of deleterious mutations that are homozygous in individual i.

As expected because of purging, there is a partial recovery in the fitness initially loss by inbreeding depression. This is due to purging reducing the inbreeding load over generations.

The following SLiM configuration file was run in SLiM 3.5:

```
// set up a simple neutral simulation
initialize() {
    setSeed(1234);
    initializeSLiMOptions(keepPedigrees = T);
    defineConstant("h", 0.0);
    defineConstant("s", -0.3);
    defineConstant("d", -0.5*s*(1.0-2.0*h));
    defineConstant("N", 25);
    defineConstant("RSCRIPT", "/usr/bin/Rscript");
    initializeTreeSeq();
    initializeMutationRate(5e-6);
    //initializeMutationType("m1", 0.5, "f", 0.0);
    initializeMutationType("m2", h, "f", s);
    //initializeGenomicElementType("g1", m1, 1.0);
    initializeGenomicElementType("g2", m2, 1.0);
    ends = c();
    rates = c();
    for (i in 0:1000) {
        initializeGenomicElement(g2, i*21, i*21);
        initializeGenomicElement(g2, i*21+1, i*21+20);
        ends = c(ends,  i*21, i*21+20);
        rates = c(rates, 0.5, 0.0);
```

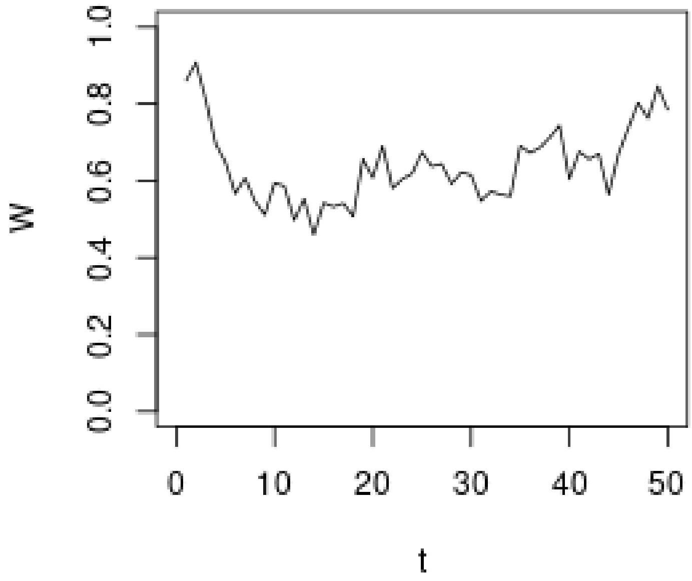

Figure 2: Change in mean fitness (W) on the bottlenecked population

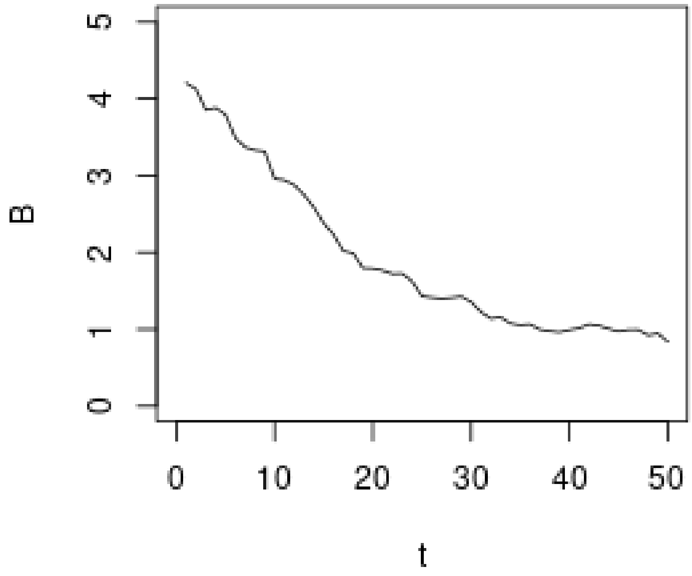

Figure 3: Decline in the inbreeding load (B) of the bottlenecked population

```
    }
    initializeRecombinationRate(rates, ends);
}

1 {
    sim.setValue("history", NULL);
    defineConstant("pngPath", writeTempFile("plot_", ".png", "")); // in QtSLiM
    if(exists("slimgui"))
        slimgui.openDocument(pngPath);
}

1 late() {
    sim.readFromPopulationFile("basepop.trees");
    p1.setSubpopulationSize(N);
}

1000:1050 late() {

    // Pedigree
    ind = p1.individuals.pedigreeID + 1;
    par = p1.individuals.pedigreeParentIDs + 1;
    generation = sim.generation-1000;

    // Inbreeding load
    mut_del = sim.mutationsOfType(m2);
    q = sim.mutationFrequencies(p1, mutations = mut_del);
    delta = 2*q*(1-q)*d;
    delta = sum(delta);
    cat("Delta: ");
    catn(delta);

    // Phenotype
    inds = sim.subpopulations.individuals;
    f = sum(sim.substitutions.mutationType == m2);
    f = (1.0+s)^f;
    p = float(N);
    b = float(N);
    for (i in 0:(N-1)) {
        w = inds[i].genome1.mutationFrequenciesInGenomes(mut_del);
        v = inds[i].genome2.mutationFrequenciesInGenomes(mut_del);
        p[i] = f;
        for (j in 0:(length(w)-1)) {
            if (w[j] & w[j] == v[j]) p[i] = p[i] * (1.0+s);
            else if (w[j] | v[j]) p[i] = p[i] * (1.0+s*h);
            if (w[j] != v[j]) b[i] = b[i] + 1.0;
        }
        b[i] = b[i] *d;
    }
    mp = mean(p);
    mb = mean(b);
    cat("Fitness: ");
    catn(mp);
    log = c(sim.getValue("history"), mb);
```

```
    sim.setValue("history", log);
    rstr = paste(c('{',
        't <- (1:' + size(log) + ')',
        'W <- c(' + paste(log, sep=", ") + ')',
        'png(width=4, height=4, units="in", res=72, file="' + pngPath + '")',
        'plot(x=t, y=W, xlim=c(0, 50), ylim=c(0, 5), type="l")',
        'dev.off()',
        '}'), sep="\n");
    scriptPath = writeTempFile("plot_", ".R", rstr);
    system(RSCRIPT, args=scriptPath);

    catn("Pedigree:");
    catn("id\tdam\tsire\tw\tb\tgeneration");
    for (i in 0:(N-1)) {
        cat (ind[i] + "\t" + par[2*i] + "\t" + par[2*i+1] + "\t");
        catn(p[i] + "\t" + b[i] + "\t" + generation);
    }
}
```
