# Supplementary material for: purgeR: inbreeding and purging in pedigreed populations
Source: Bioinformatics. 2021 Aug 18;38(2):564–5. doi: 10.1093/bioinformatics/btab599 (PMC8723146; doi:10.1093/bioinformatics/btab599)
Supplement: btab599_Supplementary_Data [file btab599_supplementary_data.zip › File S2.pdf]

purgeR: Inbreeding and purging in pedigreed populations

File S2: Performance assays

Sections below show different performance assays on simulated data sets. In general, these assays show that `purgeR v1.2` has little requirements in terms of memory usage, and works fast with pedigrees up to 10^4 individuals in total, and depths in the order of tens of generations. `purgeR` was designed to work with pedigrees of small size in mind (representative of inbred populations), and larger pedigrees may result in slow execution times, particularly when using computationally demanding functions like `ip_op()`. It is to mention that examples provided below involved extremely inbred populations that may not be representative of real populations, possibly overestimating system requirements required in practice.

Users are encouraged to request more efficient inbreeding-purging functions to analyse large pedigrees if needed (through the GitLab issue tracker: https://gitlab.com/elcortegano/purgeR/-/issues).

Execution time to estimate the inbreeding coefficient (F)

The function used in `purgeR` to estimate F is `ip_F()`. This function was selected to benchmark the use of `purgeR` in populations of different size for several reasons: 1) it is a representative function in the package, that is as well needed by other functions in the package; 2) users of `purgeR` interested in genetic purging will also be interested in estimating F, disregarding the purging model of choice; 3) functions to compute F are distributed in other packages allowing for comparison.

For testing purposes, a base population was simulated as in File S1, and a bottlenecked population or reduced size $N = 25$ was simulated for generations $t \in \{40, 400, 4000\}$ (resulting in pedigrees with total number of individuals $N_t \in \{10^3, 10^4, 10^5\}$). F was calculated with `purgeR::ip_F()`, but also with functions from the `ggroups v2.1.0` (Nilforooshan 2021) and `pedigree v1.4` (Coster 2013) packages: `ggroups::inbreed()` and `pedigree::calcInbreeding()`, respectively. Each of these functions was run ten times within a call from the `microbenchmarkCore::microbenchmark` function (Mersmann 2021) to measure mean execution times (in seconds), and once within a call from `peakRAM::peakRAM` (Quinn 2017) to measure peak RAM memory usage. All analyses were run on a Dell XPS 13 1900 laptop with processor Intel Core i7-1065G7 (1.30GHz).

`purgeR::ip_F()` showed an intermediate execution time, although its execution time was overall closer to that of `ggroups::inbreed()` than to `pedigree::calcInbreeding()`, which was the fastest option (slightly over a second to estimate inbreeding in the pedigree with size $N_t = 10^5$). `purgeR::ip_F()` estimated F in a population of $N_t = 10^4$ in an average of 3.65 seconds, but required over 30 minutes (1945.55 seconds) to estimate F with an input of $N_t = 10^5$ individuals.

In terms of memory usage, `purgeR::ip_F()` was the function with highest memory requirements, although it reached a plateau in pedigrees of the highest size (nearly 175 MiB), and as result, the function should be usable in most cases with home computers.

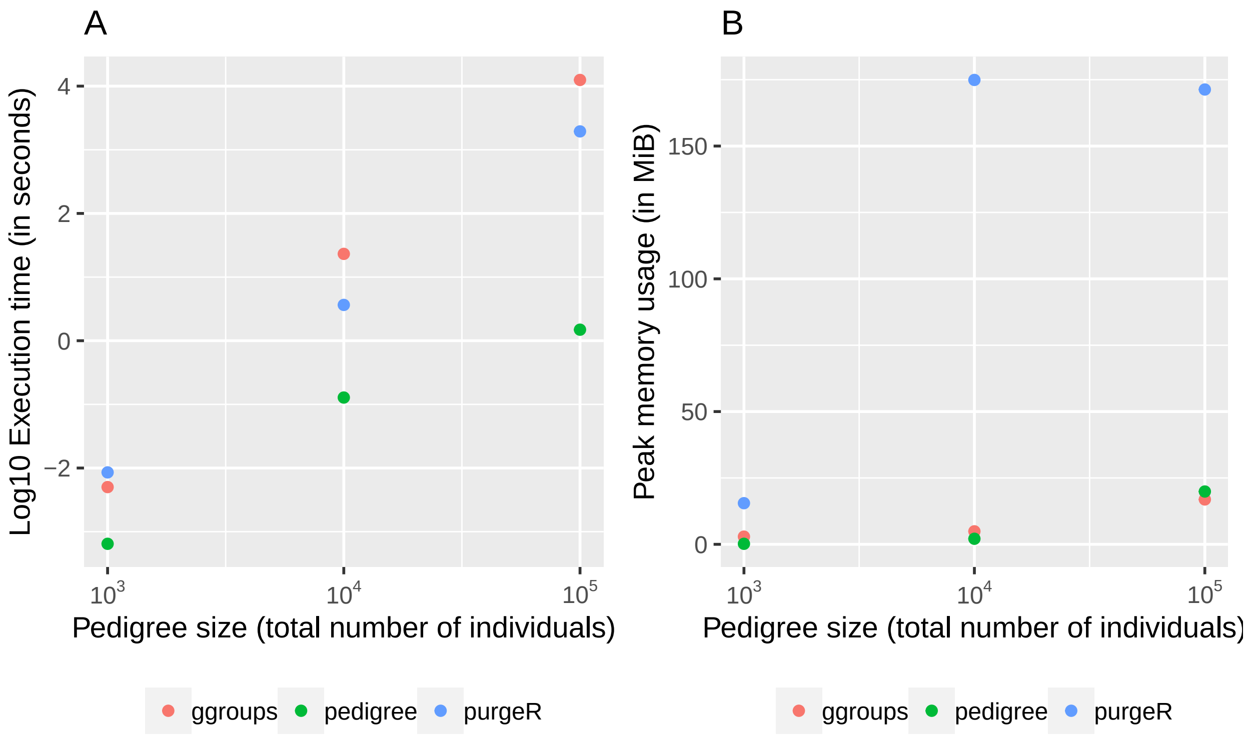

Figure 1: Execution time (A, on the left) and peak memory usage (B, on the right) when estimating F in pedigrees of increasing size with different R packages (in colors).

Estimation of purging coefficients (d)

To date, the only alternative to `purgeR` available to estimate purged inbreeding coefficients (g) is `PURGd` (García-Dorado et al. 2016). `purgeR` has many of its functions implemented from C++ functions in `PURGd`, via Rcpp code (Eddelbuettel and Francois 2011), and is therefore expected to be similar in terms of performance regarding estimates of g given a value of d.

However, there are some qualitative differences between the two softwares. The main advantage of `PURGd` over `purgeR` comes from its simplicity when it comes to estimate d, as it will request a minimal input from users and return users a table with its value. On the contrary, `purgeR` requires users to have at least some familiarity with R code, as the algorithm to estimate d needs to be user-made (examples are provided in the package vignettes).

This of course comes with the advantage that users are given more flexibility and control about how d is estimated and how regression models are handled. `purgeR` also benefits from the extensive R statistical library, introducing options to estimate d that are unavailable in `PURGd`. For example, random factors cannot be included in models in `PURGd`.

The major advantage however comes from the better goodness of fit of the models generated with `purgeR` compared to those generated in `PURGd` with default options. This is first because the algorithm implemented in `PURGd` is entirely numerical, and requires intensive exploration of the hyperspace of parameters, without guarantee to reach an optimum solution. Secondly, the convergence criterion defined in `PURGd` is highly arbitrary. By default, the program will stop running after ten iterations of numerical search if the best residual sum of squares (i.e. *likelihood*) achieved is not improved. While in simple pedigrees this might not be a problem, it is in models including environmental factors, particularly categorical variables with many levels.

To test the performance of the two tools estimating d, a toy example was generated from simulated population available in Table S1 (see code below). Three cases of study were considered: First, one without environmental factors and only $g(d)$ as regressor; Second, a more complex model including "b" as numeric variable, and "sex" as a binomial variable; Third, a model including "b" again, together with a categorical variable named "location", with six levels (please note in all cases that the models assumed do not aim to accurately estimate d, but just to explore the effect on performance of using alternative models). The purging coefficient was estimated in `PURGd` in two ways, with default options ("default"), and increasing the convergence criterion to 100 iterations ("intensive"). The intercept was estimated with the `--w0` option. `purgeR` was run using code as in the tutorial vignette (`purgeR` v1.2) , involving the use of `stats::nls()` to run a non-linear regression model as the one implemented in `PURGd` (see code below for the simpler scenario). All analyses were run 10 times on a Dell XPS 13 1900 laptop with processor Intel Core i7-1065G7 (1.30GHz).

```
library("purgeR")
library("tidyverse")
library("readxl")

bottlepop <- readxl::read_excel("Table S1.xlsx", skip = 3) %>%
  ped_rename() %>%
  dplyr::mutate(sex = rbinom(n(), 1, 0.5),
                location = sample(c("LA", "LB", "LC", "LD", "LE", "LF"), n(), TRUE),
                v = 1) %>%
  tidyr::spread(location, v, fill = 0)

# Define the range of "d" values to explore (the same accuracy = 0.01 is in PURGd)
d_values <- seq(from = 0.0, to = 0.5, by = 0.01)

# Estimate starting values for the non-linear regression call
start_values <- seq_along(d_values) %>%
    map(~ip_g(ped = bottlepop, d = d_values[[.]], name_to = "g")) %>%
    map(~dplyr::select(., -id, -dam, -sire, -generation)) %>%
    map(~glm(formula = w ~ g, data = .)) %>%
```

```r
    map("coefficients") %>%
    map(~set_names(x = ., nm = c("W0", "B")))

# Fit the non-linear model for all "d" values considered
models <- seq_along(d_values) %>%
  map(~ip_g(ped = bottlepop, d = d_values[[.]], name_to = "g")) %>%
  map2(start_values, ~nls(formula = w ~ W0 * exp(B * g), start = .y, data = .x))

# Get a summary for the best model and return RSS
RSS <- models %>% map((residuals)) %>% map(~ .x^2) %>% map_dbl(sum)
best_RSS <- min(RSS)
best_RSS
models[[which(RSS == best_RSS)]]
d_values[[which(RSS == best_RSS)]]
```

For the scenarios run here, `PURGd` never returned a better fit than `purgeR` (measures through the residual sum of squares, RSS), under the expectation of fitness following Morton et al (1956) model, as in `PURGd` (see García-Dorado et al. 2016). When `PURGd` was run with an increased number of iterations to reach convergence the goodness of fit improved, but at the cost of a much higher execution time. In all cases, running `PURGd` with default options was the quickest option, but was also the one showing the highest RSS, and the most variable one (i.e. implying low reproducibility). Note that variability in RSS means variability of the estimates of the regression coefficients, as well also of the purging coefficient estimate. `PURGd` therefore shows a strong compromise between accuracy and performance, particularly in more complex models involving more factors and levels per factor. `purgeR` on the other hand, is more consistent in terms of the quality of the fit, and the time to complete analyses. Of course, the code shown above is an example, and users are encouraged to use alternative models according to their needs.

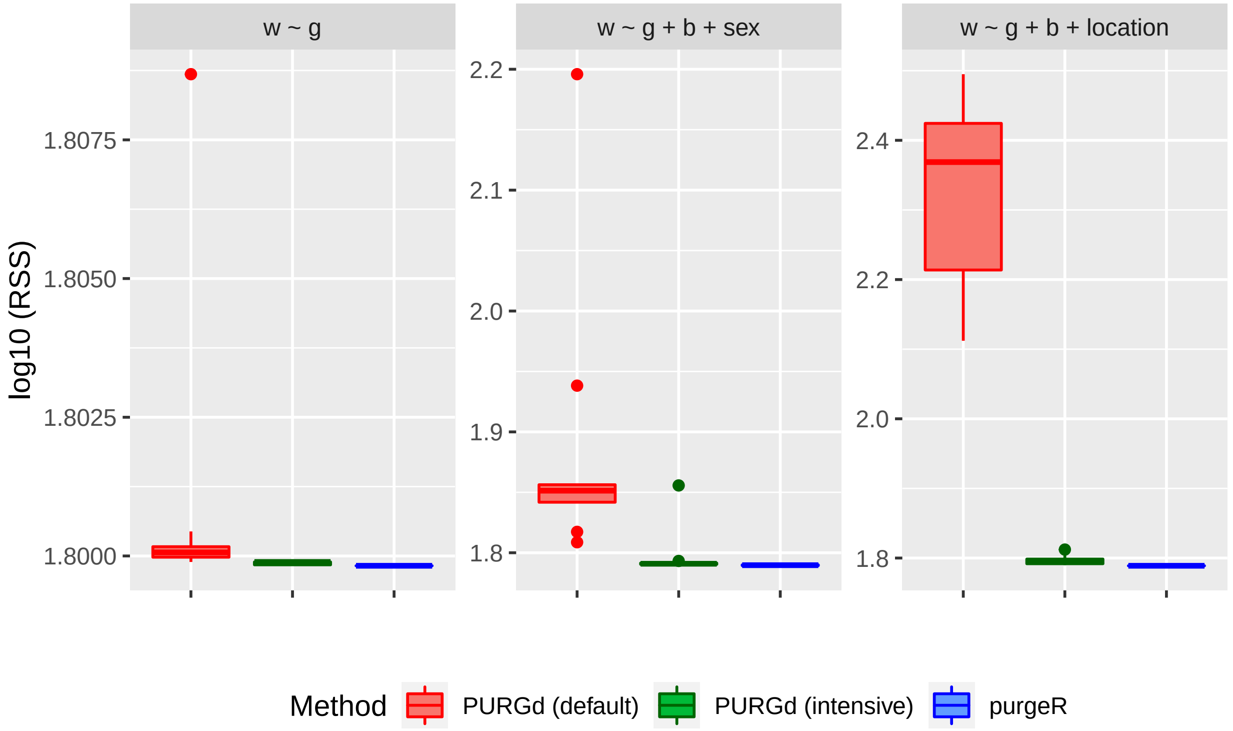

Figure 2: Log10 residual sum of squares (RSS) of the best fit while estimating d with different methods (in colors) and under different models (fitness (`w`), always as response variable, and one of more of the following factors as independent variables: purged inbreeding (`g`), inbreeding load (`b`), sex and location)

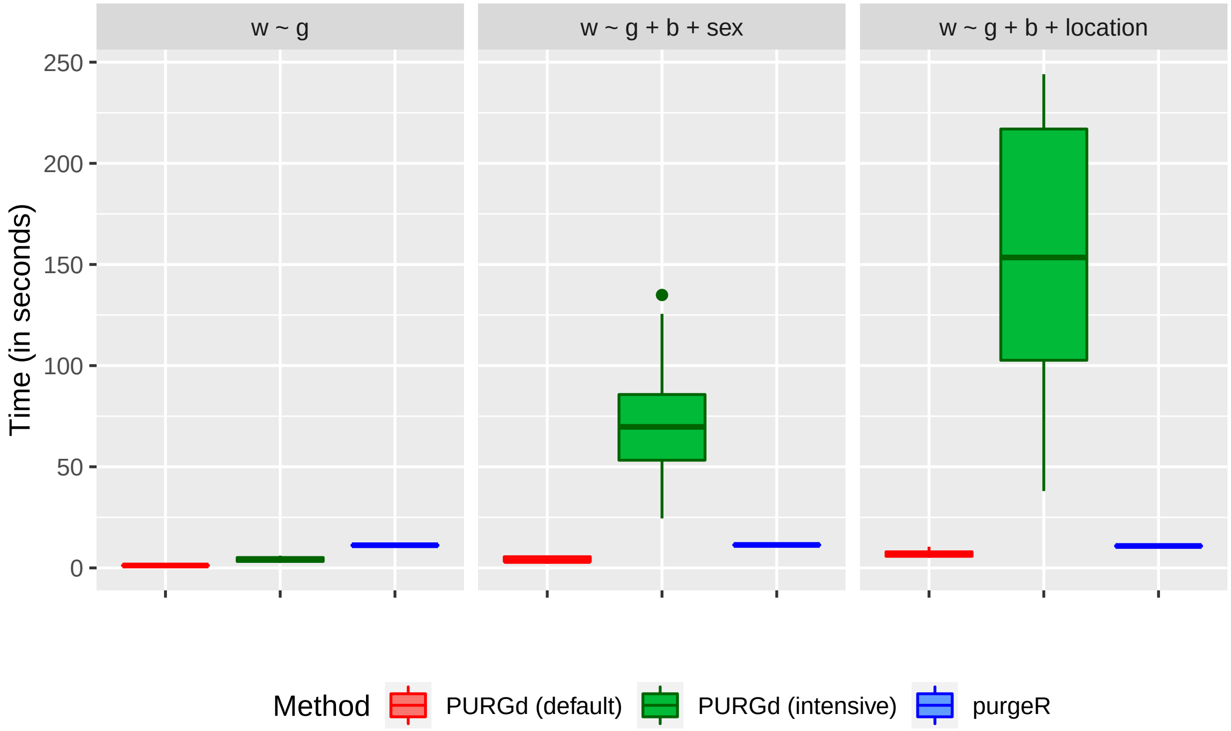

Figure 3: Execution time to estimate d with different methods (in colors) and under different models (fitness (`w`), always as response variable, and one of more of the following factors as independent variables: purged inbreeding (`g`), inbreeding load (`b`), sex and location)

Execution time and RAM usage for to obtain Opportunity of purging parameters (O and O_e)

The most computationally demanding function in `purgeR` is `ip_op()`. This is in part due to the need of the method to pre-compute a complete partial inbreeding matrix via call to `purgeR::ip_Fij(mode = "all")`, and therefore evaluate up to $N \times N \times N$ relationships.

Here, simulations with the mutation model assumed in Figure S1 are run for populations with population size $N = 25$ for $t \in \{20, 40, 60, 80, 100\}$, and $N = 100$ for $t \in \{5, 10, 15, 20, 25\}$. This is to represent two different "pedigree architectures", one with small intra-generational population size but a long track of generations recorded, and other with higher population size but few generations recorded. The function `ip_op()` was called two times with `ip_op(complex = FALSE)` and the other two with `ip_op(complex = TRUE)`. In all cases, parallelization was disabled (`ip_op(ncores = 1)` as assumed by default). All executions were queued simultaneously in the same cluster, with architecture Cluster SMP NUMA and processor Intel IA 64 Itanium 2 Montvale Dual Core 1.600MHz (6.4 Gflops).

Execution time (in minutes) was evaluated only for the `ip_op()` call, via the `base::Sys.time()` function. Peak RAM usage (in Mebibytes, MiB) was measured with `peakRAM::peakRAM()` (Quinn 2017).

For the range of parameters explored, the main factor affecting execution time is the total size of the population ($N \times t$), with not much difference between the two population architectures, differentiated by their intra-generation population size (see figure below). When the heuristic to correct O and O_e is disabled, computation time increases slightly, but not significantly.

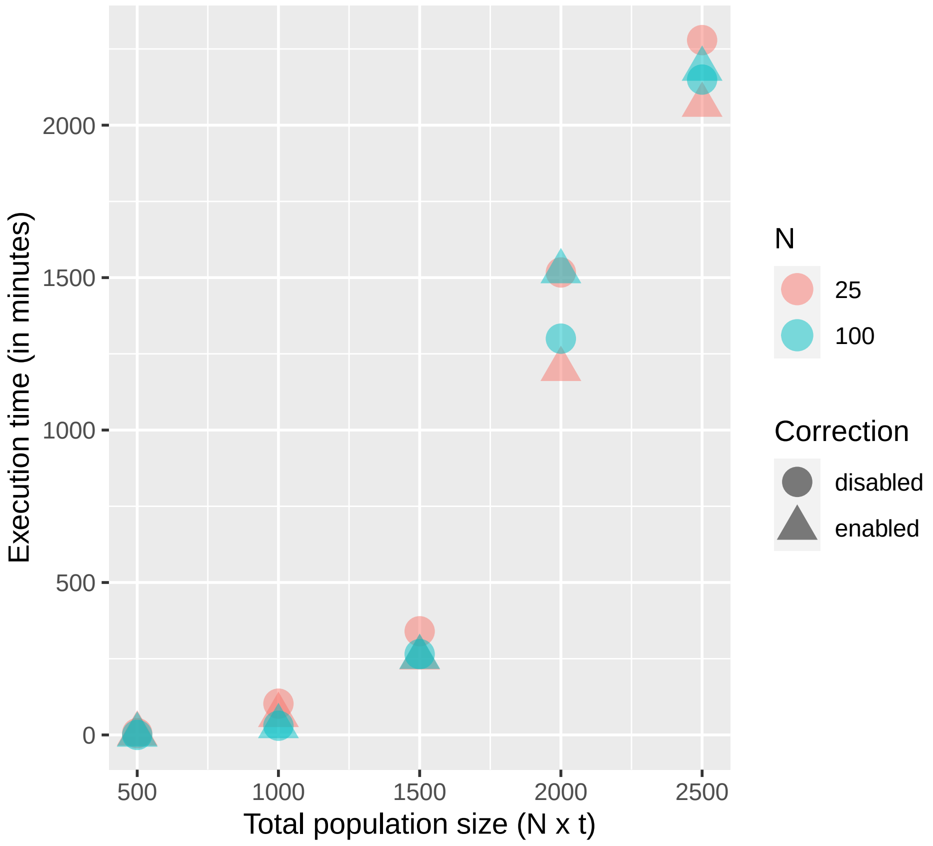

Figure 4: Execution time to run `ip_op()` with increasing pedigree size. Different number of individuals per generation is shown in colors. Circle points correspond to executions disabling the method to correct O and Oe, and triangles to executions enabling it.

This is supported by a generalized linear model (GLM) run with `lmerTest::lmer` (Kuznetsova et al. 2017). The GLM was fit with execution time in log10 scale as response variable, N, t, $N \times t$ and a binary variable for corrected or non corrected measures as fixed effects, and the number of replicate as random effect (two replicates run per case). The number of parameters in the model was reduced via backward stepwise regression using AIC as an indicator of the model likelihood. As result, N, t and $N \times t$ remained as fixed effects in the model. Results show that $N \times t$ was the most significant factor, and the only one with a positive effect on execution time:

```
profile %>%
  dplyr::mutate(Nt = N*t) %>%
  lmerTest::lmer(formula = log10(`time (min)`) ~ (1|rep) + N + t + Nt) %>%
  base::summary()
```

```
## boundary (singular) fit: see ?isSingular
```

```
## Linear mixed model fit by REML. t-tests use Satterthwaite's method [
## lmerModLmerTest]
## Formula: log10(`time (min)`) ~ (1 | rep) + N + t + Nt
##    Data: .
##
## REML criterion at convergence: 91.1
##
## Scaled residuals:
##     Min      1Q  Median      3Q     Max
## -1.8711 -0.6648  0.3506  0.6356  1.5495
##
## Random effects:
##  Groups   Name        Variance Std.Dev.
##  rep      (Intercept) 0.0000   0.0000
##  Residual             0.2433   0.4932
## Number of obs: 40, groups:  rep, 2
##
## Fixed effects:
##               Estimate Std. Error         df t value Pr(>|t|)
## (Intercept)  0.8572893  0.3554736 36.0000000   2.412   0.0211 *
## N           -0.0213508  0.0048771 36.0000000  -4.378 9.87e-05 ***
## t           -0.0258600  0.0073524 36.0000000  -3.517   0.0012 **
## Nt           0.0023501  0.0002144 36.0000000  10.963 5.03e-13 ***
## ---
## Signif. codes:  0 '***' 0.001 '**' 0.01 '*' 0.05 '.' 0.1 ' ' 1
##
## Correlation of Fixed Effects:
##    (Intr) N      t
## N  -0.857
## t  -0.776  0.905
## Nt  0.426 -0.776 -0.857
## optimizer (nloptwrap) convergence code: 0 (OK)
## boundary (singular) fit: see ?isSingular
```

As a result, the total number of individuals is the main factor to consider when evaluating the usability of `ip_op()` in a pedigree. As longer pedigrees are analyzed, execution times increase exponentially. It is to warn however against the use of the above model to fit predictions on expected execution times, given the high correlation between the variables involved. In addition, the populations simulated here are intensively inbred, involving complex relationships of ancestry (and heavy computations), and might not reflect the situation of most real populations. For example, for the data sets with $N > 1000$ included as examples in the package, computation time was about 20 minutes, which is below the values recorded for populations of with $N = 25$

and $t = 40$ (83.6 minutes on average), and $N = 100$ and $t = 10$ (29.13 minutes on average).

However, it is clear from results and figure above that populations with more than 1000 individuals will benefit from enabling parallelization options to maintain computation times short. It is also apparent that the function could become unusable for extremely large populations in the order of tens of thousands individuals (future releases will consider approaches to speed up computation).

Regarding memory usage, $N \times t$ was the only factor found to have a significant effect in a GLM assuming log10 scale for the response variable. A backward step-wise regression approach was again followed to select the best model explaining memory usage. As result, $N \times t$ was found to the the only relevant parameter ($b_{N\times t} = 2.627 \times 10^{-4} (P < 2 \times 10^{-16})$). In general, our results show that `ip_op()` (and `ip_Fij()`) maintain a low RAM usage, as the maximum value recorded was 150.7 MiB for pedigrees of 2500 individuals. It is therefore expected that pedigrees with about 3×10^4 individuals will run without issue in most home computers (assuming 8 GB of RAM memory), but users with larger pedigrees might require the use of clusters with increased RAM availability.

```
profile %>%
  dplyr::mutate(Nt = N*t) %>%
  lmerTest::lmer(formula = log10(`RAM (MiB)`) ~ (1|rep) + Nt) %>%
  base::summary()
```

```
## boundary (singular) fit: see ?isSingular
```

```
## Linear mixed model fit by REML. t-tests use Satterthwaite's method [
## lmerModLmerTest]
## Formula: log10(`RAM (MiB)`) ~ (1 | rep) + Nt
##    Data: .
##
## REML criterion at convergence: -91.3
##
## Scaled residuals:
##     Min      1Q  Median      3Q     Max
## -1.5968 -1.2314  0.1108  0.9831  1.0979
##
## Random effects:
##  Groups   Name        Variance Std.Dev.
##  rep      (Intercept) 0.000000 0.00000
##  Residual             0.003086 0.05555
## Number of obs: 40, groups:  rep, 2
##
## Fixed effects:
##              Estimate Std. Error        df t value Pr(>|t|)
## (Intercept) 1.460e+00  2.060e-02 3.800e+01   70.89   <2e-16 ***
## Nt          2.627e-04  1.242e-05 3.800e+01   21.14   <2e-16 ***
## ---
## Signif. codes:  0 '***' 0.001 '**' 0.01 '*' 0.05 '.' 0.1 ' ' 1
##
## Correlation of Fixed Effects:
##    (Intr)
## Nt -0.905
## optimizer (nloptwrap) convergence code: 0 (OK)
## boundary (singular) fit: see ?isSingular
```

References

- Coster A. (2013). pedigree: Pedigree functions. R package version 1.4. https://CRAN.R-project.org/package=pedigree
- Eddelbuettel D, Francois R. (2011). Rcpp: Seamless R and C++ Integration. Journal of Statistical Software, 40(8), 1-18. URL https://www.jstatsoft.org/v40/i08/.
- García-Dorado, A et al. (2016) Predictive model and software for inbreeding-purging analysis of pedigreed populations. G3, 6: 3593-3601.
- Gulisija D, Crow JF. (2007). Inferring purging from pedigree data. Evolution 61(5): 1043-1051.
- Kuznetsova A, Brockhoff PB, Christensen RHB (2017). “lmerTest Package: Tests in Linear Mixed Effects Models.” *Journal of Statistical Software*, *82*(13), 1-26. doi: 10.18637/jss.v082.i13 (URL: https://doi.org/10.18637/jss.v082.i13).
- Mersmann O. (2021). microbenchmark: Accurate Timing Functions. R package version 1.4-2.11. https://github.com/olafmersmann/microbenchmark/
- Morton, NE et al. (1956) An estimate of the mutational damage in man from data on consanguineous marriages. Proc. Natl. Acad. Sci. USA 42: 855–863.
- Nilforooshan MA (2021). ggroups: Pedigree and Genetic Groups. R package version 2.1.0. https://CRAN.R-project.org/package=ggroups
- Quinn T (2017). peakRAM: Monitor the Total and Peak RAM Used by an Expression or Function. R package version 1.0.2. https://CRAN.R-project.org/package=peakRAM
