# Supplementary material for: purgeR: inbreeding and purging in pedigreed populations
Source: Bioinformatics. 2021 Aug 18;38(2):564–5. doi: 10.1093/bioinformatics/btab599 (PMC8723146; doi:10.1093/bioinformatics/btab599)
Supplement: btab599_Supplementary_Data [file btab599_supplementary_data.zip › File S3.pdf]

purgeR: Inbreeding and purging in pedigreed populations

File S3: Validation of the main functions

Inbreeding, purging and population parameter functions

Functions in the package have been extensively validated by following theory and working examples by hand. However, for many of the functions included, additional validation has been performed using other informatic tools, whenever available. This is the case of functions related to inbreeding and purging parameters (prefixed with `ip_` in `purgeR`), and functions related to population parameters (prefixed with `pop_`), with the notable exception of the function for opportunity of purging (`ip_op`) that is mentioned in sections below (together with partial inbreeding). At the time of doing these validation checks, `purgeR v1.2` was used.

Inbreeding and purging

Functions for standard inbreeding (`ip_F()`) and purged inbreeding (`ip_g()`) were validated with `PURGd v2.3.2` (García-Dorado et al. 2016), and had in fact inherited their code from this software (but see notes on performance in File S2). Estimates of the inbreeding coefficient (F) also match those obtained with different software packages like `pedigree v1.4` (Coster 2013), `ggroups v2.1.0` (Nilforooshan 2021) and others.

Regarding ancestral inbreeding (F_a), `GRain v2.2` (Doekes et al. 2020) was used for validation. Note however, that contrary to `purgeR::ip_Fa()`, `GRain` uses the genedrop approach to estimate F_a by default. Validation across tools can be achieved by running high number of iterations, e.g. with `ip_Fa(genedrop = 1000000)`.

Population parameters

The main population parameter computed is the effective population size (N_e, via the `pop_Ne()` function). This parameter is computed simply from the classical formula: $N_e = \frac{1}{2\Delta F_t}$ (Falconer and Mackay 1996). Although ΔF is computed via Gutiérrez et al. (2008, 2009), it has been as well validated in populations with no overlapping generations using the more known expression $\Delta F_t = \frac{F_{t+1}-F_t}{1-F_t}$. In addition, `ENDOG v4.8` (Gutiérrez and Goyache 2005) was used to validate the use of `purgeR::pop_Ne()`.

Parameters related to the number of founders and ancestors (see `pop_Nancestors()`) were validated with tools in `PEDIG v3` (Boichard 2002). In particular, `prob_orig.f` was used to compute effective number of founders, and `segreg.f` for the number of founder genome equivalents (a high number of replicates $> 10^4$ needs to be assumed to increase reproducibility across tools).

Similarly, the number of equivalent to complete generations (`purgeR::pop_t()`) was tested with the `PEDIG` tool `n_gen.f`.

Finally, the deviation from Hardy-Weinberg equilibrium (`purgeR::pop_hwd()`) was validated with `Metapop v2.4` (López-Cortegano et al. 2019) using the `--pedigree` option to read pedigree structure data rather than molecular data.

Opportunity of purging: Pedigree example

The total (O) and expressed (O_e) opportunity of purging were first described by Gulisija and Crow (2007), and a pedigree example was provided to illustrate the computation of partial inbreeding coefficients ($F_{i(j)}$), as well as of O and O_e. This pedigree can be accessed from `purgeR::ip_op()` documentation page, or built from scratch as:

```
library("purgeR")
pedigree <- data.frame(
  id = c("M", "K", "J", "a", "c", "b", "e", "d", "I"),
  dam = c("0", "0", "0", "K", "M", "a", "c", "c", "e"),
  sire = c("0", "0", "0", "J", "a", "J", "b", "b", "d")
)
pedigree <- purgeR::ped_rename(pedigree, keep_names = TRUE)
pedigree
```

```
##   id dam sire names
## 1  1   0    0     M
## 2  2   0    0     K
## 3  3   0    0     J
## 4  4   2    3     a
## 5  5   1    4     c
## 6  6   4    3     b
## 7  7   5    6     e
## 8  8   5    6     d
## 9  9   7    8     I
```

Using the tabular method to compute partial kinship coefficients, one can observe that the same values of $F_{i(j)}$ reported in Gulisija and Crow (2007) are outputed by `purgeR::ip_Fij`. For example, $F_{I(J)} = 7/32 = 0.21875$. This is value related to row (i.e. individual) 9 below:

```
ip_Fij(pedigree, mode = "custom", ancestors = c(3L))
```

```
## Computing partial kinship matrix. This may take a while.
```

```
##             3
##  [1,] 0.00000
##  [2,] 0.00000
##  [3,] 0.00000
##  [4,] 0.00000
##  [5,] 0.00000
##  [6,] 0.25000
##  [7,] 0.12500
##  [8,] 0.12500
##  [9,] 0.21875
```

```
# Note the use of "L" here to specify integers.
# Alternatively, use as.integer(3)
```

`GRain` was also used to validate partial inbreeding coefficients relative to founders (the default use of `ip_Fij()`). Similar estimates can be obtained with `GRain`, but since this software uses a simulation-based approach, a large number of replicates is required.

Regarding measures of the opportunity of purging in the pedigree above, $O_e(I) = 1/16 = 0.0625$, as reported in the original article:

```
pedigree <- ip_F(pedigree)
ip_op(pedigree, Fcol = "Fi", compute_O = TRUE)
```

```
## Computing partial kinship matrix. This may take a while.
```

```
##   id dam sire names     Fi      O     Oe
## 1  1   0    0     M 0.0000 0.0000 0.0000
## 2  2   0    0     K 0.0000 0.0000 0.0000
## 3  3   0    0     J 0.0000 0.0000 0.0000
## 4  4   2    3     a 0.0000 0.0000 0.0000
## 5  5   1    4     c 0.0000 0.0000 0.0000
## 6  6   4    3     b 0.2500 0.0000 0.0000
## 7  7   5    6     e 0.1875 0.1250 0.0000
## 8  8   5    6     d 0.1875 0.1250 0.0000
## 9  9   7    8     I 0.3750 0.3125 0.0625
```

However, there are differences between the values of $O(I) = 0.25$ and the one reported by `purgeR`, $O(I) = 0.3125$:

The reason for the discrepancy is likely to be a typo in the original article. Let's follow the *by hand* calculation of this parameter for individual I, provided that calculation of the inbreeding coefficient (F) reported in Guliisja and Crow (2007) is the same as computed with `ip_F()`.

Following Gulisija and Crow (2007), O_i equals:

$$O_i = \sum_j \sum_k (\frac{1}{2})^{n-1} F_j$$

where the summation k is over all paths to i from ancestor j and summation j is over all inbred ancestors of i, and n is the number of individuals in the path counting j and i.

In consequence, considering that only b, d, and e are inbred ancestors of I, $O(I)$ in the pedigree example must equal the following,

$O(I) = 2 \times (\frac{1}{2})^2 F_b + (\frac{1}{2}) F_d + (\frac{1}{2}) F_e$

$O(I) = 0.125 + 0.09375 + 0.09375$

$O(I) = 0.3125$

This is the result generated by `ip_op()`. Following Guliisja and Crow (2007) example, the path from ancestor b was only counted once, leading to:

$O(I) = (\frac{1}{2})^2 F_b + (\frac{1}{2}) F_d + (\frac{1}{2}) F_e$

$O(I) = 0.0625 + 0.09375 + 0.09375$

$O(I) = 0.25$

However, this does not satisfy expression above where $(\frac{1}{2})^{n-1} F_j$ is to be looped over all inbred ancestors **and their paths** to i. In other words, the example given in Guliisja and Crow (2007) only considered one of the two paths: {b → d → I} and {b → e → I}, but the two of them had to be included, and this is accounted for in `purgeR::ip_op()`.

For the same reason, individuals d and e also have $O > 0$, since they have inbred ancestry (b).

Opportunity of purging: Simulated populations for different mutational models

The importance of O and O_e comes from their association with the individual reduction in inbreeding load. In the main text, a simple example was given to illustrate this relationship. Here, a more exhaustive set of simulations is used, covering a range of mutation models (always with fixed effects), and differently bottlenecked populations.

Populations were simulated with the same SLiM configuration files as in File S1, with the following exceptions regarding selection coefficient (s), degree of dominance (h) and mutation rate (u) parameters:

Table 1: Mutational models

s	h	u
0.01	0.0	3.5e-5
0.10	0.0	8.5e-6
0.20	0.0	6e-6
0.30	0.0	5e-6
0.40	0.0	4e-6
0.50	0.0	3.6e-6
0.60	0.0	3.4e-6
0.70	0.0	3.2e-6
0.80	0.0	2.9e-6
0.90	0.0	2.7e-6
1.00	0.0	2.6e-6
0.01	0.1	5.6e-5
0.10	0.1	3.1e-5
0.20	0.1	2.9e-5
0.30	0.1	2.8e-5
0.40	0.1	2.75e-5
0.50	0.1	2.72e-5
0.60	0.1	2.7e-5
0.70	0.1	2.68e-5
0.80	0.1	2.67e-5
0.90	0.1	2.66e-5
1.00	0.1	2.65e-5

Note that the above cases where tuned so that the value of inbreeding load (B) before bottlenecking the populations were in all cases in the range $4.3 \leq B \leq 4.5$ after rounding to the first decimal, and $B_{t=0} = 4.4$ will be assumed below.

One base population was simulated per mutational model, and ten bottlenecked populations were derived from each of them (disabling the seed parameter), with sizes $N \in \{10, 25, 50\}$. O_e was computed for each pedigree with `ip_op()` for both raw measures (without enabling the correction for complex pedigrees), and corrected measures (enabling the heuristic proposed in the main text to correct O_e).

Inbreeding load is expected to decline in relation to the normalized O_e (O_e/F, Gulisija and Crow 2007), so that $E(B_t) = B_{t=0} \times (1 - O_e/F)$. It is also expected that `ip_op()` return a similar pattern of O_e change (and $E(B_t)$) for all mutational models assumed, since the underlying method is only aware of the pedigree structure and increase in inbreeding, and not real effect of mutations. As a consequence, `ip_op()` returns similar profiles of expected decline in inbreeding load over generations, and only differentiates across demographic models, and when the heuristic to correct O_e is used.

Raw estimates of O_e without enabling the heuristic to introduce correction terms result in predictions that B declines much faster than when the correction algorithm is enabled.

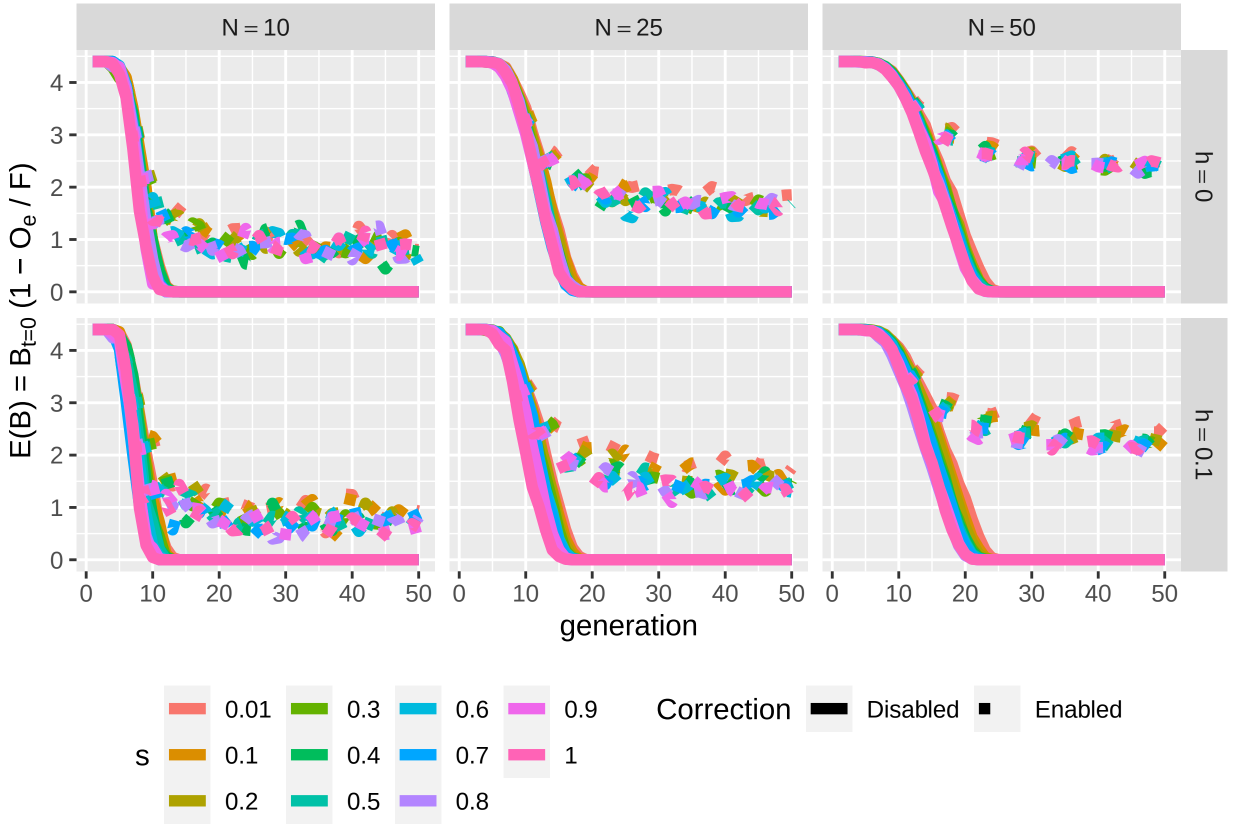

Figure 1: Expected decline of the inbreeding load (B) using corrected (dotted line) and non corrected (solid lines) estimates of Oe. Colors identify data from populations simulated under mutational models with different s (and u) parameter.

Given the above results, we next average estimations of the expected B over all values of s, and focus on how they account for the actual B decline in the simulated populations. This is shown in the figure below, where black lines show the expected decline in B conditional to enabling or not the proposed heuristic, and colored lines show the observed B decline in the different simulated populations, conditional to the value of s (and u) of the mutational model.

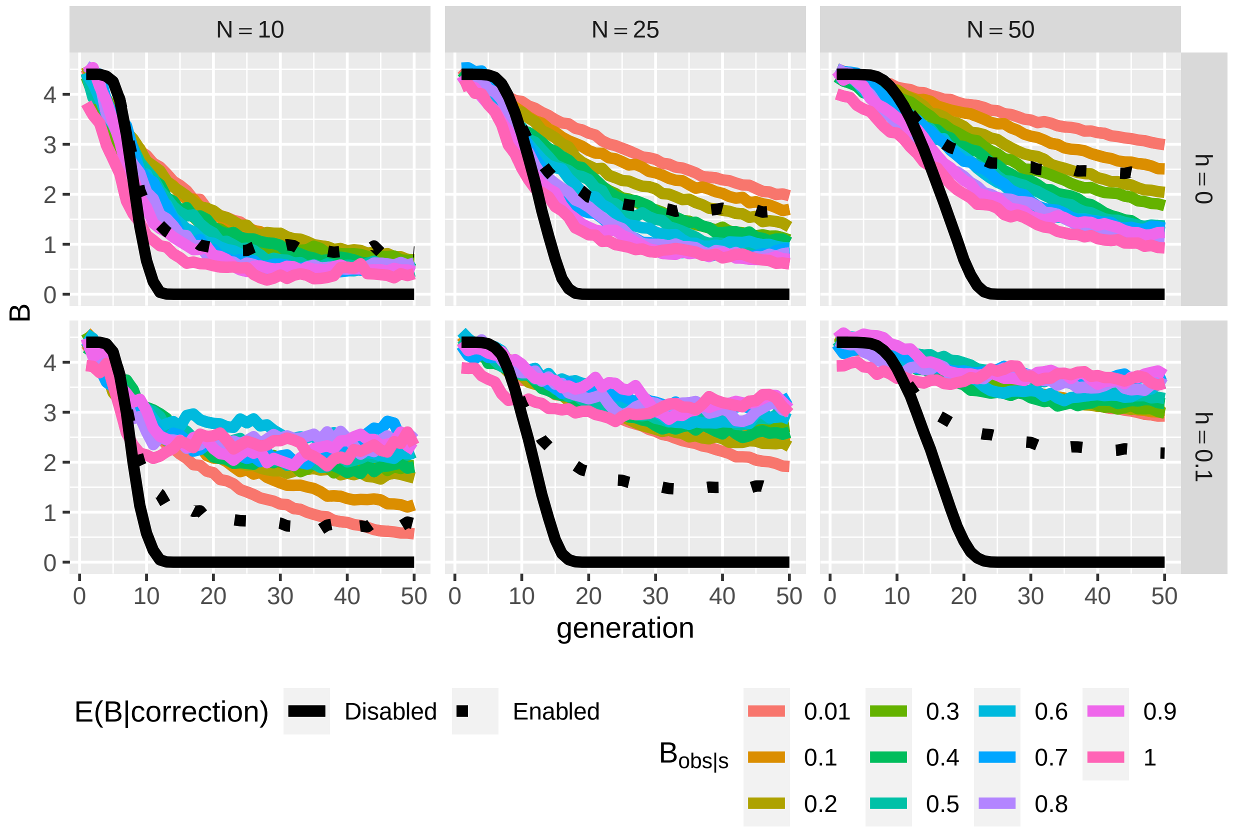

Figure 2: In black lines, expected decline of the inbreeding load (B) using corrected (dotted line) and non corrected (solid lines) estimates of Oe, averaged across mutational models. Colored lines indicate observed B across mutational models.

As shown in the figure, the highest bias (i.e. difference between observed B and the two expected B estimates) is detected in cases with partially recessive mutations ($h = 0.1$). This is expected, since Gulisija and Crow (2007) purging model assumes complete recessivity ($h = 0.0$). Absolute precision estimating B decline is never obtained from estimation of O_e neither enabling ($E(B|correction = enabled)$) or nor ($E(B|correction = disabled)$) the proposed heuristic. However, actual decline of B more frequently falls between $E(B|correction = enabled)$ and $E(B|correction = disabled)$ when the assumed mutational model related to mutations of high effect size, as assumed by Gulisija and Crow (2007).

It must be noted that examples above are intended to be illustrative and are not exhaustive (e.g. effects such as linkage disequilibrium are not explored, and simple demographic events are assumed). They do however show that both raw estimates of O_e and estimates using the heuristic proposed here are together useful to estimate a range of B decline, as long as the only the most deleterious and recessive component of the inbreeding load is assumed.

It is also worth mentioning that simulated populations here are extremely inbred, and the heuristic proposed will remove most ancestral contributions to O_e, which might explain its asymptotic behavior in figures above. This is less likely to occur in real situations , e.g. see figure below for the expected decline of B in *Ammotragus lervia* pedigree (see their documentation pages in the package to learn more about this data set). For this species, the normalized O_e increases with generations, and as for the last cohort, a narrow range between 66 % and 79 % of B ascribed to highly deleterious recessive loci is expected to be purged.

```
data(arrui)

# Note: versions of purgeR >1.2 will return both raw and corrected estimates in
# a single run
arrui <- arrui %>%
  purgeR::ip_F() %>%
  purgeR::ip_op(complex = FALSE, Fcol = "Fi") %>%
  dplyr::rename(Oe_raw = Oe) %>%
  purgeR::ip_op(Fcol = "Fi") %>%
  dplyr::mutate(species = "A. lervia") %>%
  purgeR::pop_t() %>%
  dplyr::mutate(t = plyr::round_any(t, 1))
```

```
arrui %>%
  dplyr::group_by(species, t) %>%
  dplyr::summarise(Fi = mean(Fi),
                   Oe = mean(Oe),
                   Oe_raw = mean(Oe_raw),
                   nOe = Oe/Fi, nOe_raw = Oe_raw/Fi) %>%
  ggplot(aes(x = t)) +
  geom_point(aes(y = nOe, color = "enabled")) +
  geom_point(aes(y = nOe_raw, color = "disabled")) +
  geom_line(aes(y = nOe, color = "enabled")) +
  geom_line(aes(y = nOe_raw, color = "disabled")) +
  facet_grid(. ~ species) +
  scale_y_continuous(expression(paste(O["e"], " / F", sep = "")), limits = c(0, 1)) +
  scale_x_continuous("Equivalent to complete generations", breaks = c(0,1,2,3,4,5,6,7)) +
  scale_color_manual("Correction", values = c(enabled  = "blue", disabled = "red")) +
  theme(strip.text = element_text(size = 12, face = "italic"),
        legend.position = "bottom")
```

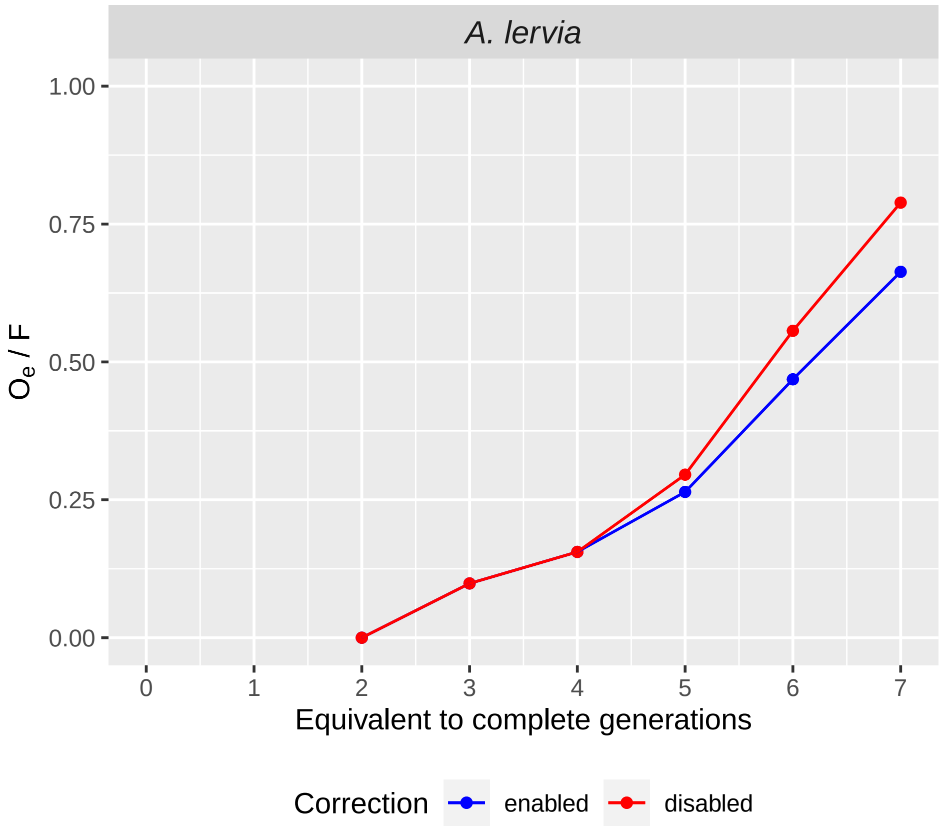

Figure 3: Change in normalized opportunity of purging in *Ammotragus lervia* pedigree over generations. In colors, estimates using corrected (blue) and non corrected (red) estimates of *Oe*.

References

- Ballou JD. (1997). Ancestral inbreeding only minimally affects inbreeding depression in mammalian populations. J. Hered., 88, 169-178.
- Coster A (2013). pedigree: Pedigree functions. R package version 1.4. https://CRAN.R-project.org/package=pedigree.
- Doekes HP et al. (2020). Revised calculation of Kalinowski's ancestral and new inbreeding coefficients. Diversity, 12, 155.
- Falconer DS and Mackay TFC. (1996). Introduction to Quantitative Genetics. 4th edition. Longman, Essex, U.K.
- García-Dorado A et al. (2016) Predictive model and software for inbreeding-purging analysis of pedigreed populations. G3, 6: 3593-3601.
- Gulisija D, Crow JF. (2007). Inferring purging from pedigree data. Evolution 61(5): 1043-1051.
- Gutiérrez D and Goyache F. (2005). A note on ENDOG: a computer program for analysing pedigree information. Journal of Animal Breeding and Genetics, 122: 172-176
- Gutiérrez JP, et al. (2008). Individual increase in inbreeding allows estimating effective sizes from pedigrees. Genet. Sel. Evol. 40: 359-378. Gutiérrez JP, et al. (2009). Improving the estimation of realized effective population sizes in farm animals. J. Anim. Breed. Genet. 126: 327-332.
- Haller BC, Messer PW. (2019). SLiM 3: Forward genetic simulations beyond the Wright-Fisher model. Mol. Biol. Evol., 36, 632-637.
- López-Cortegano E., et al. (2019). Metapop2: Re-implementation of software for the analysis and management of subdivided populations using gene and allelic diversity. Molecular Ecology Resources 19(4): 1095-1100.
- Nilforooshan, MA (2021). ggroups: Pedigree and Genetic Groups. R package version 2.1.0. https://CRAN.R-project.org/package=ggroups
