# Supplementary material for: purgeR: inbreeding and purging in pedigreed populations
Source: Bioinformatics. 2021 Aug 18;38(2):564–5. doi: 10.1093/bioinformatics/btab599 (PMC8723146; doi:10.1093/bioinformatics/btab599)
Supplement: btab599_Supplementary_Data [file btab599_supplementary_data.zip › File S4.pdf]

purgeR: Inbreeding and purging in pedigreed populations

File S4: R code used for figures in the manuscript

Estimate all values of genealogical inbreeding (standard, ancestral and purged)

```
bottlepop <- readxl::read_excel("Table S1.xlsx", skip = 3)
bottlepop <- bottlepop %>%
  purgeR::ped_rename() %>%
  purgeR::ip_F() %>%
  purgeR::ip_Fa(Fcol = "Fi") %>%
  purgeR::ip_g(d = 0.15, Fcol = "Fi")
```

Opportunity of purging

```
# Inbreeding load in the starting generation
bottlepop <- bottlepop %>% dplyr::rename(t = generation)
B <- bottlepop %>% dplyr::filter(t == 1) %>% .$b %>% mean()

# Computation of opportunity of purging parameters
# Note: versions of purgeR >1.2 will return both raw and corrected estimates in
# a single run
bottlepop <- bottlepop %>%
  purgeR::ip_op(Fcol = "Fi", ncores = 4, complex = FALSE) %>%
  dplyr::rename(Oe_raw = Oe) %>%
  purgeR::ip_op(Fcol = "Fi", ncores = 4, complex = TRUE)
```

```
## Computing partial kinship matrix. This may take a while.

## Computing partial kinship matrix. This may take a while.
```

```
# Inbreeding load decline
Bdecline <- bottlepop %>%
  dplyr::filter(dam!=sire | dam == 0) %>%
  dplyr::mutate(nOe = ifelse(Fi == 0.0, 0.0, ifelse(Oe > Fi, 1.0, Oe/Fi)),
                nOe_raw = ifelse(Fi == 0.0, 0.0, ifelse(Oe_raw > Fi, 1.0, Oe_raw/Fi)),
                eb = B*(1.0-nOe),
                eb_raw = B*(1.0-nOe_raw)) %>%
  dplyr::group_by(t) %>%
  dplyr::summarise(bh = sd(b),
                   bl = quantile(b, probs = 0.025),
                   b = mean(b, na.rm = TRUE),
                   eb = mean(eb, na.rm = TRUE),
                   eb_raw = mean(eb_raw, na.rm = TRUE)) %>%
  ggplot +
  geom_point(aes(x=t, y= b), color = "red", pch = 21) +
  geom_errorbar(aes(x = t, ymin = b-bh, ymax = b + bh), color = "red", width = 0.2) +
  geom_line(aes(x=t, y=eb, linetype = "enabled"), color = "blue") +
  geom_line(aes(x=t, y=eb_raw, linetype = "disabled"), color = "blue") +
```

```
  annotate("text", x = 35, y = 4.5,
           label= "Observed B", color = "red", size = 5, hjust = 0) +
  annotate("text", x = 35, y = 4,
           label = expression(paste(B["t=0"], " (1 - ", O["e"], " / F)", sep = "")), color = "blue", si
  scale_y_continuous(expression(paste("Inbreeding load (", italic(B), ")", sep = ""))) +
  scale_x_continuous(expression(italic("t")), breaks = c(0, 10, 20, 30, 40, 50)) +
  scale_linetype_manual("Correction", values = c(enabled = "solid", disabled = "dotted")) +
  ggtitle(label = "A") +
  theme(panel.background = element_blank(),
        axis.line = element_line(size = 0.1),
        axis.title = element_text(size = 18),
        axis.text = element_text(size = 15),
        legend.title = element_text(size = 20),
        legend.text = element_text(size = 15),
        legend.position = "bottom",
        plot.title = element_text(size=25))
Bdecline
```

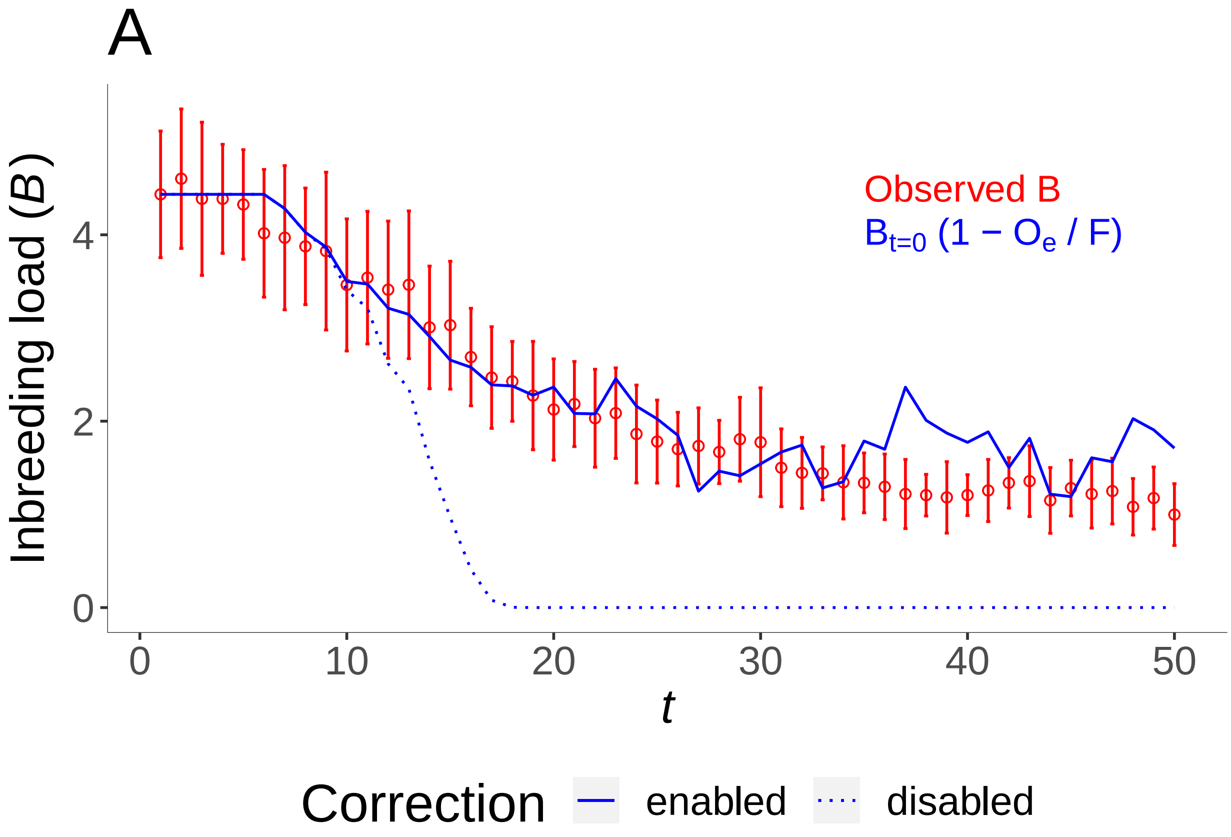


```
# BOe
BOe <- bottlepop %>%
    dplyr::filter(dam != sire | dam == 0) %>%
    ggplot() +
    geom_point(aes(x = Fi, y = Oe, fill = b), pch = 21, size = 6, alpha = 0.8) +
    geom_abline(intercept = 0, slope = 1, linetype = "dashed") +
    scale_fill_gradient2("B", low = "blue", high = "red",
                         midpoint = 1.8, mid = "white", space = "Lab") +
```

```
    scale_y_continuous(expression(paste("Expressed opportunity of purging (",
                                        italic(O["e"]), ")",
                                        sep = ""))) +
    scale_x_continuous(expression(paste("Inbreeding coefficient (",
                                        italic("F"), ")",
                                        sep = "")), limits = c(0, 0.8)) +
    ggtitle(label = "B") +
    theme(panel.background = element_blank(),
          axis.line = element_line(size = 0.1),
          axis.title = element_text(size = 18),
          axis.text = element_text(size = 15),
          legend.title = element_text(size = 20),
          legend.text = element_text(size = 15),
          plot.title = element_text(size=25))

BOe
```

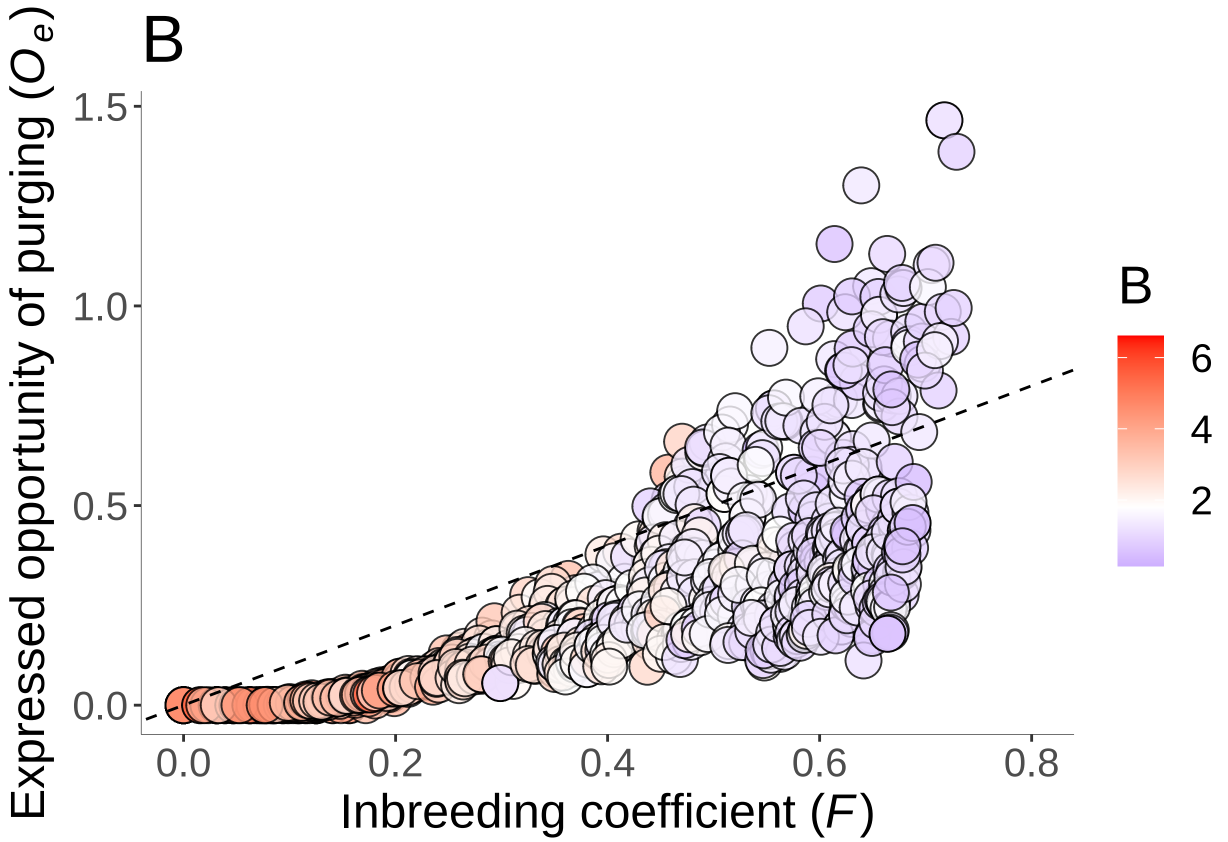


```
# G <- grid.arrange(Bdecline, BOe, ncol = 2)
# ggsave(G, filename = "F1.tiff", width = 16, height = 6, dpi = 320, device = "tiff")
```
